# Usnic acid improves memory impairment after cerebral ischemia/reperfusion injuries by anti-neuroinflammatory, anti-oxidant, and anti-apoptotic properties

**DOI:** 10.22038/ijbms.2020.43280.10165

**Published:** 2020-09

**Authors:** Sohaila Erfani, Tahereh Valadbeigi, Nahid Aboutaleb, Naser Karimi, Ali Moghimi, Mehdi Khaksari

**Affiliations:** 1 Department of Biology, Faculty of Science, Ilam University, Ilam, Iran; 2 Physiology Research Center and Department of Physiology, Faculty of Medicine, Iran University of Medical Sciences, Tehran, Iran; 3 Department of Physiology, Faculty of Medicine, Iran University of Medical Sciences, Tehran, Iran; 4 Department of Biology, Faculty of Science, Razi University of Kermanshah, Kermanshah, Iran; 5 Rayan Center for Neuroscience and Behavior, Department of Biology, Faculty of Science, Ferdowsi University of Mashhad, Mashhad, Iran; 6 School of Medicine, Shahroud University of Medical Sciences, Shahroud, Iran

**Keywords:** Apoptosis, Cerebral ischemia, Lichen secondary-metabolites, Neuroinflammation, Spatial memory

## Abstract

**Objective(s):**

Cerebral ischemia/reperfusion causes complex pathological mechanisms that lead to brain tissue damage. Usnic acid is a lichen secondary metabolite that has many different biological properties including anti-inflammatory and anti-oxidant activities. Therefore, the objective of the current study was to investigate the neuroprotective effects of usnic acid on apoptotic cell death, neuroinflammation, anti-oxidant enzyme activities, and oxidative stress levels after transient cerebral ischemia/reperfusion.

**Materials and Methods:**

Forty-two male Wistar rats were randomly assigned to three groups (sham, ischemia/reperfusion, and ischemia/reperfusion+usnic acid). Ischemia was induced by 20 min occlusion of common carotid arteries. Injection of usnic acid (25 mg/kg, intraperitoneally) and saline was done at the beginning of reperfusion time. Morris water maze was applied to assess spatial memory. The protein expression amount was measured using immunohistochemical and immunofluorescence staining. Spectrophotometric assay was performed to determine the levels of anti-oxidant enzymes.

**Results:**

Usnic acid significantly reduced caspase-3, glial fibrillary acidic protein- positive and ionized calcium-binding adaptor molecule 1-positive cells (*P<*0.001) and enhanced spatial memory disorders (*P<*0.05) due to brain ischemia. In addition, treatment with usnic acid improves effects in the antioxidant system following cerebral ischemia (*P<*0.05).

**Conclusion:**

Our findings indicate that usnic acid has neuroprotective properties, which possibly is applicable as a promising candidate for cerebral injuries caused by ischemia.

## Introduction

Lichens are some of the most important sources of biologically active compounds that have been applied in traditional medicine owing to their precious therapeutic properties (1). These organisms have many different biological activities including anti-inflammatory, anti-oxidant, antipyretic, analgesic, anticancer, antimicrobial, antidiabetic, and wound healing properties (2). The most commonly used genus of lichen is *Usnea* *Dill. ex Adans*, which has applications in medicine worldwide. For example, *Usnea longissima* *Ach.* is a type of lichen species that is extensively used for injury treatment caused by bone fractures and inflammatory skin eruptions (3).

Macrolichens are sources of phenolic secondary substances (e.g. phenols, quinones, dibenzofurans, depsides, depsones, depsidones, γ-lactones, pulvinic acid derivatives, and xanthones). Dibenzofuran of usnic acid (2,6-Diacetyl-7,9-dihydroxy-8,9 b-dimethyl-1,3 (2H,9bH)-dibenzofurandione) is known as a lichen secondary metabolite which is generated to form a yellow pigment in several lichen species (4). G. Amo *et al.* indicated that usnic acid has inhibitory effects on reactive oxygen species (ROS) synthesis due to hydrogen peroxide in a human astrocytoma cell line, and this anti-oxidant ability leads to improvement of cell viability (5). Moreover, usnic acid shows anti-inflammatory impact by reduction of pro-inflammatory cytokine expression, like tumor necrosis factor-alpha (TNF-α), interleukin-6 (IL-6) and IL-1β, also increases IL-10 expression as an anti-inflammatory cytokine in LPS-stimulated inflammatory cellular model (6). Odabasoglu *et al*. showed that usnic acid has gastroprotective effects via increasing superoxide dismutase (SOD), glutathione peroxidase (GPx), and glutathione (GSH) activities and decreasing lipid peroxidation (LPO) level in indomethacin-induced gastric ulcer tissues in an animal model (7).

Cerebral ischemia/reperfusion (I/R) is the third most common factor of death in developed countries which induces considerable mortality and disability worldwide. It causes complex pathological mechanisms that can lead to tissue damage (8). Excitotoxicity, oxidative stress, inflammation, and apoptosis are the most important pathogenic processes that are associated with cerebral ischemia (9). Therefore, factors that could exhibit anti-oxidant, anti-apoptotic, and anti-inflammatory characteristics are helpful to treat cerebral ischemia (10). Cerebral I/R induces the neuroinflammation process, which is indicated by astroglial and microglial activation, cytotoxic agent secretion such as nitric oxide (NO), ROS, inflammatory cytokines, and matrix metalloproteinases enzymes. Such agents cause blood-brain barrier breakdown and postpone the death of neurons (11).

Astrocytes are known as crucial brain mediators capable of releasing several pro-inflammatory factors following ischemia, including intermediate-filament and glial fibrillary acidic protein (GFAP) (12). Among mice, GFAP protein loss has been attributed to increased susceptibility to ischemic tissue injury after middle cerebral artery occlusion. Moreover, the previous findings have indicated that GFAP is up-regulated by reactive astrocytes in several neurodegenerative situations, like ischemia, and for this reason it is abundantly used as an alternative indicator of brain damage after ischemia (13). 

The expression of ionized calcium-binding adaptor protein-1 (Iba-1) (actin-binding protein (17 kDa))can be particularly seen in all microglia. It has proven crucial for microglia adjustment, particularly in activated microglia it has extensively been used as an immunohistochemical marker (14). Thus, in various studies, the level of GFAP and Iba-1 expression is applied to detect activated astrocyte and microglia (15, 16). The pyramidal cells of the CA1 area of the hippocampus are more susceptible to ischemia compared to other areas of the brain. Several studies have reported that apoptosis promotes overexpression of Bax and caspase-3 genes in the CA1 area after global ischemia (17, 18). Furthermore, it has been demonstrated that the hippocampus is crucial in impaired spatial memory following brain ischemia (19). Considering all the above-mentioned facts about molecular mechanisms of brain ischemia and usnic acid properties, we investigated the neuroprotective impacts of usnic acid after brain I/R. 

## Materials and Methods

***Animals and drugs***

Forty-two adult male Wistar rats (250-300 g) were procured from Tehran Pasteur Institute and kept in a room (22-24 ^°^C; humidity: 45-50%) under a regular 12/12 L/D cycle (08:00-20:00) with free access to water and food. Experiments were performed in the light phase. Daily handling of cases was performed for 5 days (5 min/day) before the experiments. Helsinki Declaration principles were followed in performing the experiments. For injection, usnic acid (Sigma Aldrich, 329967, Germany) was dissolved in DMSO (20).

***Experimental groups***

Animals were randomly assigned to three separate groups (14 animals for each group), including sham, ischemia/reperfusion, and ischemia/reperfusion+usnic acid groups. Animals in ischemia/reperfusion+usnic acid group received a single IP injection of usnic acid (25 mg/kg) at the beginning of reperfusion time. To induce cerebral I/R, the common carotid arteries occlusion (CCA) was performed. In the sham group, the same surgical procedure was performed without obstruction of their common carotid arteries. 

***Transient global cerebral I/R model***

Induction of transient global cerebral I/R model was performed as described previously (21). Before beginning the brain ischemia surgery, anesthetization was done using IP injection of ketamine (50 mg/kg) and xylazine (10 mg/kg). Bilateral common carotid arteries were exposed followed by dividing from the carotid sheath and vagus nerves. Yashargil aneurism micro clips were applied for occlusion of the common carotid artery within 20 min. When the occlusion time was finished, the clips were removed for immediate establishing of reperfusion period (48 hr). Restoration of blood flow was visually observed. Animals’ rectal temperature was kept at 36.5±0.5 ^°^C along with experimental period by the feedback regulatory heating system. At the end of surgery, animals were put in individual home cages with free access to food and water for 7 days.

***Morris water maze (MWM) task***

The assessment of spatial learning and memory was done using MWM to evaluate cognitive impairment (22). It was embedded in an illuminated room and external clues were placed in different parts of the room and were preserved in the same position throughout training and probe trial.

***Spatial training test***

This test was performed 3 days after exertion of the cerebral ischemia model. Training of the rats was done for finding the hidden submerged platform through four consecutive days via four trials and an interval of 20 sec each 3 to 6 days following inducing the ischemia model. The platform was placed in the northwest quadrant followed by putting animals in the water facing the wall from one random quadrant. In all trials, the animals could swim to find and climb the platform. They were kept on the platform within 30 sec. When they failed to discover the platform in 60 sec they were assisted by the researcher to find the platform. The escape latency (time spent to find the platform) was noted through a video/tracking device. The average results related to four trials was calculated.

***Spatial probe test***

To calculate the 60 sec spatial probe trial test, 24 hr after the last training (seven days following exertion of ischemia), we removed the platform from the tank followed by recording the time spent in the target quadrant (training test).

***Tissue preparation for staining***

Following the memory test, seven animals (half of animals in each group) were subjected to anesthesia as well as transcardiac perfusion by 0.9% saline and following that 4% paraformaldehyde in 0.1 M of phosphate buffer (pH 7.4) (23). Afterward, brain tissues were extracted, the same fixative was used for post fixation process throughout 3 days followed by paraffin embedding of brains. Then, coronal portions (7 µm) were taken according to the Paxinos atlas (between 3.3 mm and 4.2 mm posterior to bregma) by a microtome using staining techniques.

***Measurement of caspase-3 immunoreactivity***

For evaluation of caspase-3 activation, immunofluorescence staining was done using tissue parts (16). Incubation (20 min/62 ^°^C) and rehydration by descending series of alcohol was performed; in the next step, 10% hydrogen peroxide in methanol within 10 min was used for reducing activity of endogenous peroxidase. Afterward, tissue sections were washed in Tris buffer (pH 7.4), followed by retrieving antigens through autoclaving for 11 min in citrate buffer (pH 6). The segments were blocked by 10% normal goat serum for an hour and then incubation was done with anti-caspase-3 antibody (rabbit antibody against rat Abcam, UK) as a primary antibody overnight (4 ^°^C). Following washing in PBS and incubation by anti-rabbit secondary antibody, the sections were fluorochrome- conjugated (Abcam, UK) within a two hour period in the dark to be able to see that the antigens were done. Then, counterstaining process with 4′,6-diamidino-2-phenylindole (DAPI) in PBS within 5 min to label the nucleus was performed. After washing, the fluorescence signals from the right hippocampal CA1 region achieved by each slide were evaluated using a fluorescence microscope (Labomed, USA, 400×). The positive cells as well as total number of cells were calculated expressed by the rate of caspase-3-positive cells compared with total cells.

***Immunohistochemical staining***

For evaluation of astrocyte and microglia activation, immunohistochemical staining was carried out on tissue sections via antibodies to GFAP and Iba-1, respectively. At first, the deparaffinization process by incubation (60 ^°^C/30 min), clarification by xylene and rehydration by subtraction of alcohols was done. Then 10% H_2_O_2_ in methanol was applied for blocking endogenous peroxidase function for 10 min. After washing in Tris buffer, retrieval process of antigen was performed by application of autoclaving in citrate buffer (C_6_H_5_Na_3_O_7_·2H_2_O, pH 6) for 11 min. Following washing in PBS, 1% fetal bovine serum (FBS) in 0.3% TritonX-100 was applied for the blocking process and then overnight incubation with primary antibody (Abcam, UK) was carried out at 4 ^°^C. Favorable dilution was 1/100. In the next step, incubation with goat polyclonal secondary antibody (HRP) (Abcam, UK) was carried out for 30 min at room temperature by adding 3, 3′-diaminobenzidine (DAB, Sigma, USA) for antigen detection. Counterstaining was done using hematoxylin (Sigma) to enable visualization under the microscope (24). Photomicrographs of the sections were provided through light microscopy. By using of light microscopy (LABOMED USA, magnification 400×) photomicrographs of the sections were prepared. The number of GFAP and Iba-1-positive cells was calculated along the right hippocampal CA1 region (400×). Whole counting methods were evaluated blindly. 

***Biochemical assessment***

Seven rats from each group (half of animals in each group) were anesthetized by ketamine (80 mg/kg) and xylazine (10 mg/kg), then the rats’ brains for the biochemical assessment were removed and the hippocampal samples were homogenized in RIPA buffer containing protease inhibitors. we used the supernatant for enzyme evaluation after 20 min of centrifugation at 3000 g (4 ^°^C) (25).

***Assessment of MDA levels***

Calculation of malondialdehyde (MDA) as an indicator for detection of lipid peroxidation products was done in the hippocampus specimens with the MDA assay kit (ZellBio GmbH, Germany). Homogenization process of prepared tissues was performed on ice-cold 1.15% KCl within 2 min. Calculation of MDA levels was done according to formation of thiobarbituric acid reactive substance technique. Cold 10% trichloroacetic acid (1 ml) and 2 ml of 10% thiobarbituric acid were blended with homogenized tissues followed by heating (100 ^°^C/ 1 hr). Then the room temperature was set for the solution and then it was centrifuged, the precipitate was discarded and the pink color supernatant was transferred into a microplate. Assessment of reaction mixture absorbance was carried out at 535 nm using a microplate reader (ELx800, BioTek, USA). The MDA level (µM) was determined based on the standard curve.

***Assessment of glutathione (GSH) and superoxide dismutase (SOD) concentrations***

GSH and SOD concentrations were assessed using the instructions expressed in GSH and SOD assay kit (ZellBio GmbH, Germany). Homogenization and centrifugation processes of the collected tissues was done following adding PBS (100 mM, pH 7.4). Afterward, collection of supernatants, SOD and GSH interacted with chromogen reagent, and the absorption rates were read via a microplatereader (ELx800, BioTek, USA), respectively at 420 and 412 nm for calculating SOD (U/ml) and GSH (mM) levels, according to a specific formula.

***Statistical analysis***

To determine whether data distribution is normal, the Kolmogorov-Smirnov test was used. One-way ANOVA was applied for assessing the differences between the groups. For statistical significant differences, Scheffe’s *post hoc* test was employed for determining the differences. *P*≤0.05 was regarded as significant. Data analysis was done using SPSS 16.0 software (SPSS Inc., Chicago, IL, USA).

## Results

***Spatial learning and memory of animals in MWM***

*The effects of usnic acid treatment on the escape latency (time to find the platform in trials in MWM)*

**Figure 1:**
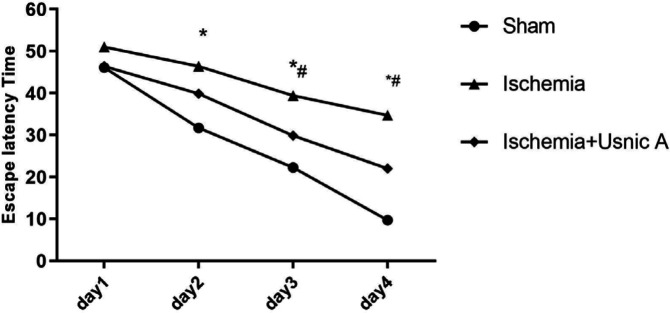
Escape latency during trial days in the Morris water maze task for different groups of rats

MWM results showed a significant difference between all groups in escape latency to find the platform in trials. The ischemia group showed more time for reaching the platform during trials rather than the sham group (*P*≤0.001). Also, in ischemic rats that received usnic acid, the time to reach the platform during the trials was significantly reduced compared with the ischemic group (*P*<0.05, Figure 1).

*The usnic acid treatment effects on the time spent in the target zone in probe trial in MWM*

**Figure 2:**
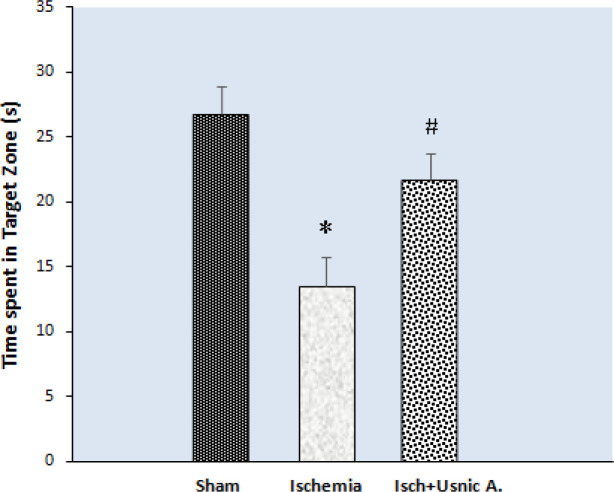
Time spent in the target zone in probe trial day in the Morris water maze

MWM results indicated a significant difference between groups in the time spent in the target zone during probe trials. Those in the ischemia group spent less time in the target zone (13.53 sec±5.42) compared with the sham group (26.77 sec±5.24) (*P*≤0.01). Ischemic rats treated with usnic acid indicated an increase in the time spent in the target zone (21.71 sec±4.99) compared with the ischemia group (*P*<0.05, Figure 2). 

***Usnic acid decreased the I/R-related caspase-3 activation***

**Figure 3:**
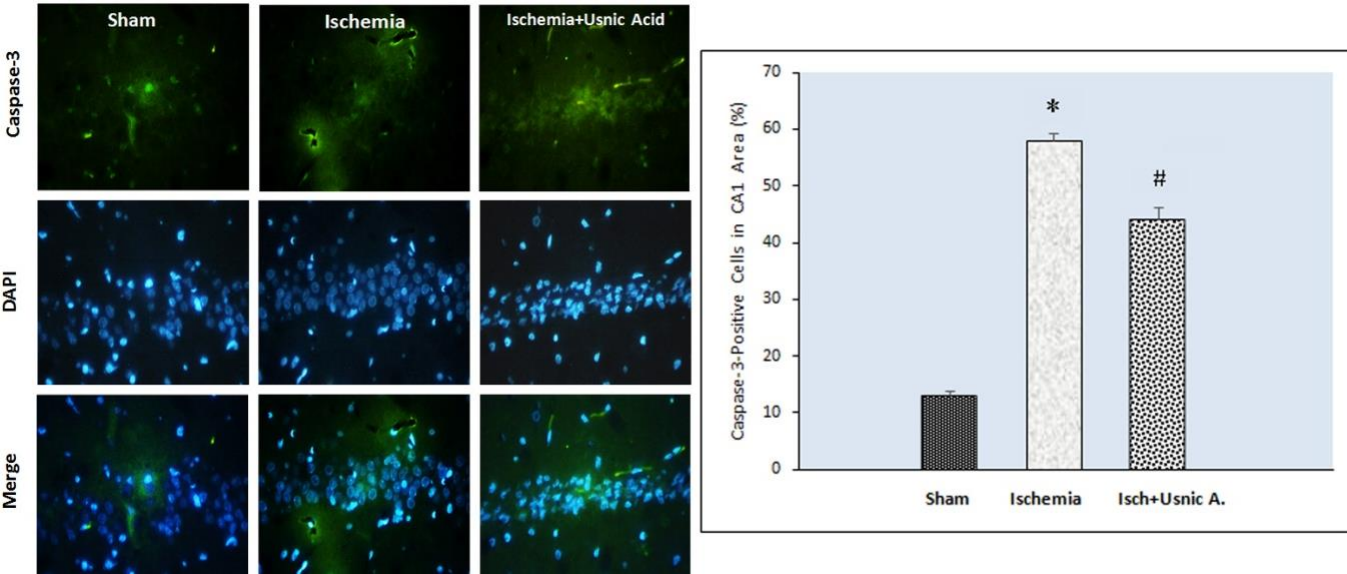
Left: Photomicrographs of caspase-3 immunofluorescence staining in the hippocampal CA1 region in different groups of following transient global cerebral ischemia in male rats. Caspase-3-stained (green) and DAPI-stained (blue) in the hippocampal segments (×400). Right: Usnic acid treatment impacts on the active caspase-3-positive cell rates in the CA1 region of hippocampus after ischemia. Usnic acid treatment significantly decreased the I/R-caused caspase-3 activation

The immunofluorescence staining findings related to caspase-3 activation showed a significant difference in all groups regarding active caspase-3-immunopositive cell rate in the CA1 area of the hippocampus. There was a significant increase in active caspase-3-positive cells in ischemia group (58.14%±3.02) in comparison with the sham-operated (13%±2.08) group (*P*<0.001). Usnic acid-treated group showed significantly lower active caspase-3-positive cells (44.14%±5.34) compared with the ischemic rats (*P*<0.001, Figure 3). 

***Usnic acid decreased GFAP protein levels after ischemia***

**Figure 4:**
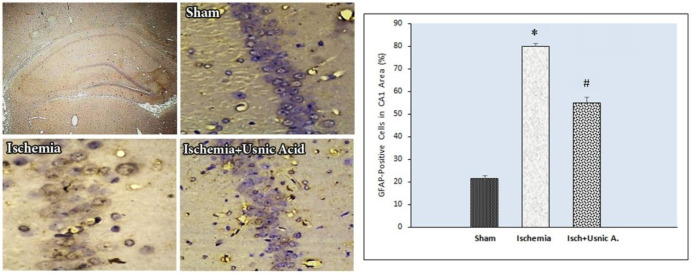
Left: Photomicrographs of immunohistochemical staining of Glial fibrillary acidic protein (GFAP) in the hippocampal CA1 region in different groups of following transient global cerebral ischemia (×400). Right: Usnic acid treatment impacts on the GFAP amounts in the CA1 region of hippocampus after ischemia. Treating with usnic acid significantly lowered an increase in GFAP levels caused by cerebral I/R

Immunohistochemical staining data belonging to GFAP protein showed an immunohistochemical statistically significant difference in GFAP-positive cell rates in the CA1 area of the hippocampus in groups. There was a slight GFAP expression in the sham-operated group (21.43%±3.78). Also, GFAP-positive cells were enhanced in ischemia group (80%±3.16) compared with the sham group (*P*<0.001). In the usnic acid-treated group, the GFAP-positive cell rate was decreased (54.86%±7.33) compared with the ischemia group (*P*<0.001, Figure 4).

***Usnic acid decreased the Iba-1 protein concentrations following ischemia***

**Figure 5:**
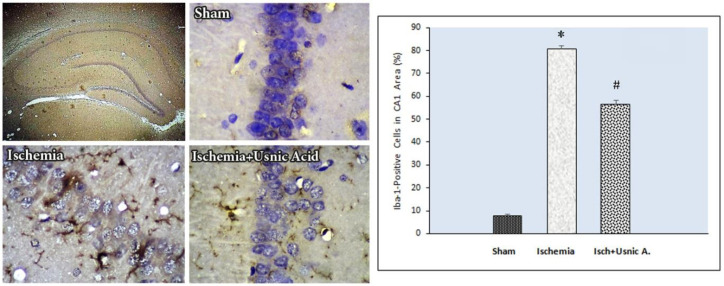
Left: Photomicrographs of immunohistochemical staining of Iba-1 in the hippocampal CA1 region in different groups of following transient global cerebral ischemia (×400). Right: Usnic acid treatment impacts on the Iba-1 amounts in the CA1 region of hippocampus after ischemia. Treating with usnic acid significantly lowered an increase in Iba-1 levels caused by cerebral I/R

Immunohistochemical staining results of Iba-1protein indicated a statistically significant difference in Iba-1-positive cell rate in the CA1 region of the hippocampus in all groups. There was a slight expression in Iba-1 in the sham-operated group (7.86%±1.86). In addition, the Iba-1-positive cell rate was increased in the ischemic group (81%±3.51) compared with the sham group (*P*<0.001). In usnic acid-treated group, there was a decrease in Iba-1-positive cell rate (56.57%±4.31) compared with the ischemic group (*P*<0.001, Figure 5).

***Treating with usnic acid caused a reduction in MDA level and an elevation in SOD and GSH activities in rat model of cerebral ischemia***

**Figure 6:**
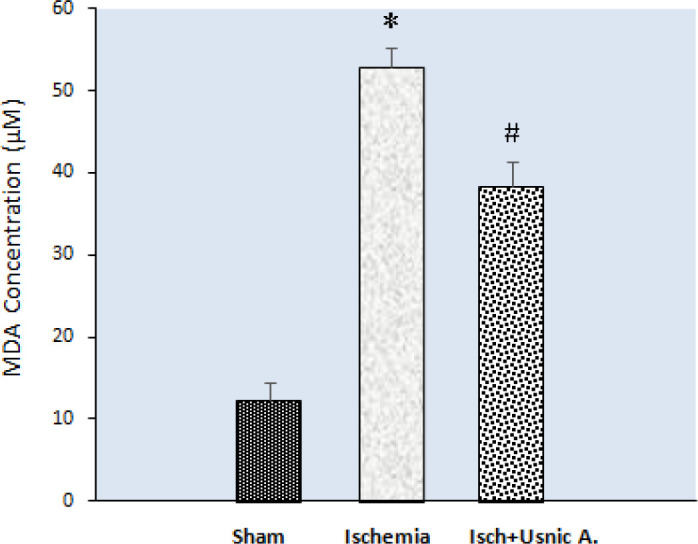
Effect of usnic acid treatment on malondialdehyde (MDA) concentration in the hippocampus after cerebral ischemia

The analysis of biochemical data related to MDA concentrations of the hippocampal tissue showed that in the ischemia group MDA levels (52.92 µM±5.46) were higher compared with the sham group (12.34 µM±5.03, *P*<0.001). Decreased MDA levels in usnic acid treatment group was seen compared with the ischemia group ((38.27 µM±7.53, *P*<0.01). Based on the findings, usnic acid is effective in inhibiting the lipid peroxidation process (Figure 6).

**Figure 7:**
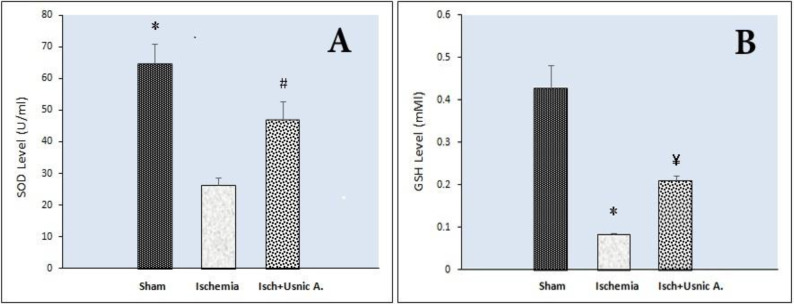
Effect of usnic acid treatment on Superoxide dismutase (SOD) (A) and glutathione (GSH) (B) levels in the hippocampus after cerebral ischemia

 Furthermore, a considerable decrease in the SOD activity was discovered in the ischemic animals (26.37 U/ml±5.9) rather than the sham group (64.67 U/ml±15.04) (*P*<0.001). In ischemia group, which had received usnic acid, the SOD activity level increased (47.04 U/ml±14.03) compared with the ischemic rat group (*P*<0.05) (Figure 7). 

Moreover, GSH amounts were decreased in the ischemia group (0.08 U/ml±0.01) compared with the sham group (0.43 U/ml±0.14) (*P*<0.001). In ischemic animals that had received usnic acid, the GSH activity level increased (0.21 U/ml±0.03) compared with the ischemia group (*P*<0.05, Figure 7). 

* Significantly different compared with sham group (*P<*0.001)

^#^Significantly different compared with ischemia+usnic group (*P<*0.05)

* Significantly different compared with sham group (*P<*0.01)

^#^ Significantly different compared with ischemia group (*P<*0.05)

* Significantly different compared with sham group (*P<*0.001)

^#^ Significantly different compared with sham and ischemia groups (*P<*0.001)

* Significantly different compared with sham group (*P<*0.001)

^#^ Significantly different compared with sham and ischemia groups (*P<*0.001)

* Significantly different compared with sham group (*P<*0.001)

^#^ Significantly different compared with sham and ischemia groups (*P<*0.001)

* Significantly different compared with sham group (*P<*0.001)

^#^ significantly different compared with sham (*P<*0.001) and ischemia groups (*P<*0.05)

* Significantly different compared with sham and usnic acid groups (*P<*0.001)

^#^ significantly different compared with ischemia group (*P<*0.05)

¥ significantly different compared with sham (*P<*0.001) and usnic acid groups (*P<*0.05)

## Discussion

The current study for the first time provided remarkable evidence about usnic acid, as a novel neuroprotective agent in treatment of cerebral ischemia injury in an animal model. The obtained results showed that usnic acid administration significantly improved memory impairment following transient global cerebral ischemia. Usnic acid not only could reduce activation rate of caspase-3 as a key apoptosis mediator in the CA1 pyramidal cells but also decrease the number of Iba-1 and GFAP-positive cells as neuroinflammation factors after cerebral ischemia. Moreover, our results revealed that usnic acid has significant effects on the inhibition of lipid peroxidation and it remarkably improves the anti-oxidant system by increasing the SOD and GSH enzyme levels following cerebral ischemia in an animal model.

 The hippocampus, especially the pyramidal cells of CA1 region, has been shown to be sensitive and susceptible to ischemic insults (26). During brain injury, neuronal loss and apoptosis process occur as continuous delayed neurodegeneration in the hippocampus (27). Hippocampus is critical for spatial memory as well as navigation (28). Thus, the memory deficits after cerebral ischemia can be linked to the hippocampus function deficiencies (29). The main factors for neuronal apoptosis are inflammation, free radicals, calcium imbalance, and excitotoxicity (17). Caspase-3 is known as a vital enzymatic mediator that has an important role in both external and internal apoptosis routes. Inhibiting active caspase-3 results in cellular protective state against oxidative stress (30). The oxidative stress process is the important inducer of apoptosis, which arises from alteration in the activity of the cytochrome chain. This process results in the reduction of ATP production and enhancement of ROS formation in hypoxia conditions (31).

The results of Fernández-Moriano *et al*. (2017) are in agreement with our findings that usnic acid has cytoprotective properties against cytotoxicity models related to H_2_O_2_ in central nervous system-like cells by decreasing ROS generation, lipid peroxidation levelsm and activation of pro-apoptotic protein (i.e., caspase-3) (32). Another study reported that treatment with usnic acid lead to inhibition of oxidative stress and inflammatory responses in an acute model of lung injury in mice followed by increased SOD and GSH activity and reduction of inflammatory chemokine expression including TNF-α, IL-6, IL-8, and macrophage inflammatory protein-2 (MIP-2) (33).

It is demonstrated that the increase of some cytokines including IL-6, IL-1β, and TNF-α in ischemia models are associated with pathways that participate in apoptotic neuronal death (34). The increase of IL-1β enhances TNF-α level in a synergistic way and these inflammatory cytokines promote the neuroinflammation process and brain ischemia damage.

Inhibition of inflammatory response reduces infarct size and neuronal ischemia injury (35, 36). A study showed that administration of poly-ε-caprolactone microsphere polymers containing usnic acid reduced significantly IL-1β, TNF-α, and NO levels in carrageenan-induced paw edema in a rat model, results of which were similar to our findings (37).

## Conclusion

Our results show that treatment with usnic acid significantly reduces CA1 cell death via caspase-3 activity reduction, and attenuates neuro-inflammation mediated microglial and astroglial activation. In addition, usnic acid improves the anti-oxidant defense system in the hippocampus and spatial memory disorders following neuronal damages by ischemia. Therefore, considering the above-mentioned promising effects of usnic acid in pathologic situations, usnic acid possibly is a new natural therapeutic compound for neurodegenerative disorders like cerebral I/R.
